# Acute maternal stress in pregnancy and cardiovascular risk factors in adolescent offspring: a birth-cohort study

**DOI:** 10.1093/ije/dyag111

**Published:** 2026-07-23

**Authors:** Moshe Shay Ben-Haim, Salomon Israel, Iaroslav Youssim, Hodaya Gabay, David S Siscovick, Orly Manor, Ora Paltiel, Ronit Calderon-Margalit, Yechiel Friedlander, Uri Pinchas Dior, Hagit Hochner

**Affiliations:** Braun School of Public Health, Faculty of Medicine, The Hebrew University of Jerusalem, Jerusalem 91120, Israel; Department of Psychology, Faculty of Social Sciences,The Hebrew University of Jerusalem, Jerusalem 91905, Israel; Braun School of Public Health, Faculty of Medicine, The Hebrew University of Jerusalem, Jerusalem 91120, Israel; Braun School of Public Health, Faculty of Medicine, The Hebrew University of Jerusalem, Jerusalem 91120, Israel; New York Academy of Medicine, New York, NY 10029, United States; Braun School of Public Health, Faculty of Medicine, The Hebrew University of Jerusalem, Jerusalem 91120, Israel; Braun School of Public Health, Faculty of Medicine, The Hebrew University of Jerusalem, Jerusalem 91120, Israel; Braun School of Public Health, Faculty of Medicine, The Hebrew University of Jerusalem, Jerusalem 91120, Israel; Braun School of Public Health, Faculty of Medicine, The Hebrew University of Jerusalem, Jerusalem 91120, Israel; Department of Obstetrics and Gynecology, Hadassah Ein Kerem Medical Center and Faculty of Medicine, Hebrew University of Jerusalem, Jerusalem 91120, Israel; Braun School of Public Health, Faculty of Medicine, The Hebrew University of Jerusalem, Jerusalem 91120, Israel

**Keywords:** perinatal stress, cardiovascular outcomes, offspring health, developmental origins of health and disease (DOHaD), cardiovascular risk, epidemiology

## Abstract

**Background:**

Cardiometabolic risk factors are major contributors to poor health outcomes and premature mortality. While the effect of prenatal exposure to stress on cardiometabolic outcomes has been examined, the findings remain inconsistent across studies, and even more so with regard to the timing of exposure. In the present study, we aimed to explore the association of short-term prenatal exposure to the acute stress of the 1967 Six-Day War with cardiovascular and anthropometric risk factors in adolescent offspring.

**Methods:**

Using a population-based birth cohort, we examined data from 61 237 offspring born in Jerusalem during 1964–76, linked to their military-draft records at age 17 years. These provided information on those exposed to acute prenatal stress and the timing of exposure within the pregnancy (*N* = 2471) as well as on height, weight, heart rate (HR), and blood pressure (BP) in adolescence. Linear regression models estimated the associations between prenatal stress and measurements at age 17 years, controlling for potential confounders.

**Results:**

Prenatal exposure to acute stress was associated with increased systolic blood pressure (SBP) and diastolic blood pressure (DBP) [SBP: *B *= 1.85, 95% confidence interval (CI): 1.15, 2.56; DBP: *B *= 1.89, 95% CI: 1.40, 2.38] and a decreased HR (*B *= –1.39, CI: –1.88, –0.91) but not with anthropometric outcomes. Furthermore, gestational age at exposure modified these associations: the associations with DBP and mean arterial pressure tended to increase and with HR to decrease the later the exposure occurred during the pregnancy.

**Conclusion:**

Findings suggest that acute maternal stress during pregnancy is associated with cardiovascular risk factors in offspring in adolescence and is modulated by the timing of the exposure.

Key Messages

- We investigated whether exposure to prenatal war-induced acute maternal stress is associated with cardiovascular risk factors in offspring aged 17 years.
- Acute maternal stress was linked to higher blood pressure and lower heart rate in offspring, and the strength of these associations changed according to the timing of the stress during the pregnancy.
- These findings highlight the critical impact of short-term maternal stress on offspring cardiovascular health, emphasizing the importance of further exploring underlying mechanisms, potentially leading to public health and preventive programs.

## Introduction

Early-life exposures have been implicated as important contributors to the pathophysiology of chronic diseases [1]. Specifically, psychosocial stressors, ranging from childhood adversities to natural or man-made disasters, were shown to affect physical and mental health throughout the life course [2, 3]. Moreover, intrauterine exposure to maternal psychosocial stress is associated with behavioral and metabolic outcomes in offspring [4, 5]. Associations of prenatal stress with long-term cardiometabolic outcomes are of particular interest given that poor cardiovascular health is a leading cause of morbidity and mortality (reviewed in [6, 7]). However, studies that examined the impact of various maternal stressors on offspring cardiovascular outcomes have provided conflicting results. While some studies demonstrate positive associations between maternal pregnancy distress or stress hormones and offspring blood pressure (BP) [8, 9], other studies show no or only a minimal impact of stress on the offspring [10] or even negative associations [11, 12]. These contradicting findings can be attributed to varying definitions of stress and the timing of exposure during pregnancy, overlooking sex-specific associations or circumstances that co-exist with the stress. For example, women with high levels of anxiety and perceived stress during pregnancy may also develop risky health behaviors such as smoking or poor dietary habits [13] that influence offspring growth and development [14, 15]. Furthermore, health risks in offspring may depend on the specific critical period within the pregnancy during which an exposure occurred, described extensively for famine (reviewed in [16]) and, to a lesser extent, for psychological stress as well. For example, prenatal exposure to maternal stress due to bereavement was associated with increased childhood overweight in one study [17] and the risk of ischemic heart disease up to middle age in another study when measured during the third trimester [18]. Another study estimating general perceived stress via questionnaires administered during the second trimester identified an increase in childhood BP in the offspring [19]. A summary of the findings and methodological differences is provided in Supplementary Table S1.

Lastly, studies of prenatal exposures to stress, including famine, have demonstrated sex-specific effects on offspring cardiovascular health [20–22]. For example, maternal prenatal cortisol levels were positively associated with the 10-year chronic heart disease risk at a mean age of 42 years among female offspring only [22]. It has therefore been suggested that the role of offspring biological sex should be considered when examining the effects of prenatal maternal stress (reviewed in [23]).

While assessing the health effects and exact timing of heterogeneous and potentially chronic psychological stressors can be elusive, this study utilizes a uniform stress-inducing acute exposure to address some of these concerns. The Jerusalem Perinatal Study (JPS) birth cohort was underway in Jerusalem in June 1967 when the city briefly experienced the Arab–Israeli Six-Day War. Thus, the prenatal exposure under study is a well-defined short-lived severe maternal psychosocial threat. Extensive perinatal data collected at birth were used to disentangle the effect of war-induced acute prenatal stress from other parental and offspring confounders. We compared anthropometric and BP measurements at age 17 years between males and females prenatally exposed to the war in different trimesters with those who were born prior to or conceived after the war. Our study hypothesis was that offspring health outcomes in late adolescence would be associated with war-induced maternal stress during pregnancy and that these effects may differ by offspring biological sex and the timing of the stress during the pregnancy.

## Methods

### Study population

This study was based on data from the JPS—a population-based cohort that includes all 92 408 births to residents of West Jerusalem from 1964 to 1976 [24]. Data included demographic and socioeconomic information, medical conditions of the mother during the current and previous pregnancies, and offspring birth weight. Detailed information on data collection has been previously described [25]. Military service in Israel is compulsory for young adults of Jewish descent; therefore, at age 17 years, the majority of adolescents are required to undergo medical examinations to determine eligibility for service. Offspring’s identity numbers enabled data linkage with the Israeli military-draft records containing information on anthropometry and BP [25]. Military-draft data were available for 81% of the male offspring and 52% of the female offspring, totaling 61 237 individuals (see Supplementary Figure S1). The lower coverage of military-draft data for females is a result of exemption from military examinations and service for women who declare practicing ultra-orthodox religious observance. Ultra-orthodox men, however, are not exempt from military examinations, yet some are later exempt from service. Comparison of participants from the original cohort with and without army data is provided in Supplementary Table S2.

### Study variables

#### Primary exposure

In June 1967, Jerusalem briefly experienced shelling and war, known as the Arab–Israeli Six-Day War. Prenatal exposure to acute maternal psychosocial stress was defined as experiencing the war *in utero*, as previously described [26, 27]. While the war itself lasted for 6 days, from 5 to 10 June, anxiety started in Israel on 19 May following prewar actions and escalated during preparations for war over the next 2 weeks [28]. The population of Jerusalem endured the greatest trauma when, in the first 3 days of the war, the city was heavily bombarded. Following the end of the war on the sixth day, the Israeli population returned to their normal life without disruptions on 11 June. As a result of its limited time, the war was primarily a severe psychological stressor that did not affect the environment or nutritional security. In this study, we used 19 May to 11 June as the war period for defining prenatal exposure to war-induced maternal stress or restricted only to the 6 days of the war in a sensitivity analysis.

#### Outcomes

Anthropometric and cardiovascular measurements in offspring at age 17 years were carried out by military medical officers using standard protocols. Measurements included standing height, body weight, and heart rate (HR). Systolic BP (SBP) and diastolic BP (DBP) were measured once in the right arm, in a sitting position, by using a mercury sphygmomanometer [25].

#### Covariates

Demographic and socioeconomic characteristics of parents were obtained from the JPS dataset as reported in birth certificates or maternity-ward logbooks at the offspring birth. A detailed description of the study variables and scales is provided in the Supplementary methods (available as Supplementary data at *IJE* online).

### Statistical analyses

Detailed description of the statistical analyses is provided in the Supplementary methods (available as Supplementary data at *IJE* online).

## Results

Of 61 237 JPS offspring with military-draft record data for age 17 years, 2471 were prenatally exposed to acute maternal stress induced by the war. Table 1 shows the characteristics of the sample by prenatal exposure to acute stress. The baseline characteristics of the groups were broadly similar. Supplementary Table S3 presents the unadjusted means of the outcome variables by the stress exposure and offspring biological sex and Supplementary Table S4 presents the unadjusted means of those born prior to or conceived after the war.

**Table 1:** Sample characteristics by prenatal exposure to war-induced acute maternal stress.

Variable	Categories/scale	Unexposed (*n* = 58 766)	Prenatal exposure to acute stress		
			1 (*n* = 675)	2 (*n* = 878)	3 (*n* = 918)
**Sex (%)**	Male	62.0	63.1	59.6	63.0
**Birth weight (kg)**	Continuous	3.29 ± 0.50	3.31 ± 0.48	3.31 ± 0.48	3.29 ± 0.49
**Paternal age (years)**	Continuous	31.5 ± 6.6	32.1 ± 7.0	31.4 ± 6.3	31.8 ± 6.6
**Maternal age (years)**	Continuous	27.6 ± 5.5	28.0 ± 5.9	27.6 ± 5.7	27.6 ± 5.9
**Paternal origin (%)**					
	Israel	13.6	12.7	13.4	12.1
	North Africa	22.6	24.4	22.8	22.1
	West Asia	33.2	34.8	36.8	36.5
	West Europe/Other	30.6	28.1	27.0	29.3
**Maternal origin (%)**					
	Israel	12.6	11.3	13.2	13.5
	North Africa	24.4	25.5	23.9	24.9
	West Asia	31.5	36.3	37.2	36.5
	West Europe/Other	31.5	26.9	25.7	25.1
**Paternal education (years)**	Continuous	11.2 ± 4.2	10.1 ± 4.6	10.2 ± 4.6	10.2 ± 4.2
**Maternal education (years)**	Continuous	10.5 ± 4.2	9.1 ± 4.5	9.5 ± 4.4	9.3 ± 4.4
**SES (%)**					
	Low	25.3	35.4	29.6	31.8
	Medium	41.4	34.4	40.6	41.2
	High	33.3	30.2	29.8	27.0
**Years since immigration**	Continuous	14.9 ± 7.5	13.9 ± 7.2	13.8 ± 6.2	13.9 ± 6.3

Values are expressed as percentages or mean ± SD. Only offspring with measurements of BP and obesity-related traits at age 17 years are included. SES denotes socioeconomic status categorized based on father’s occupation.

**Figure 1:**
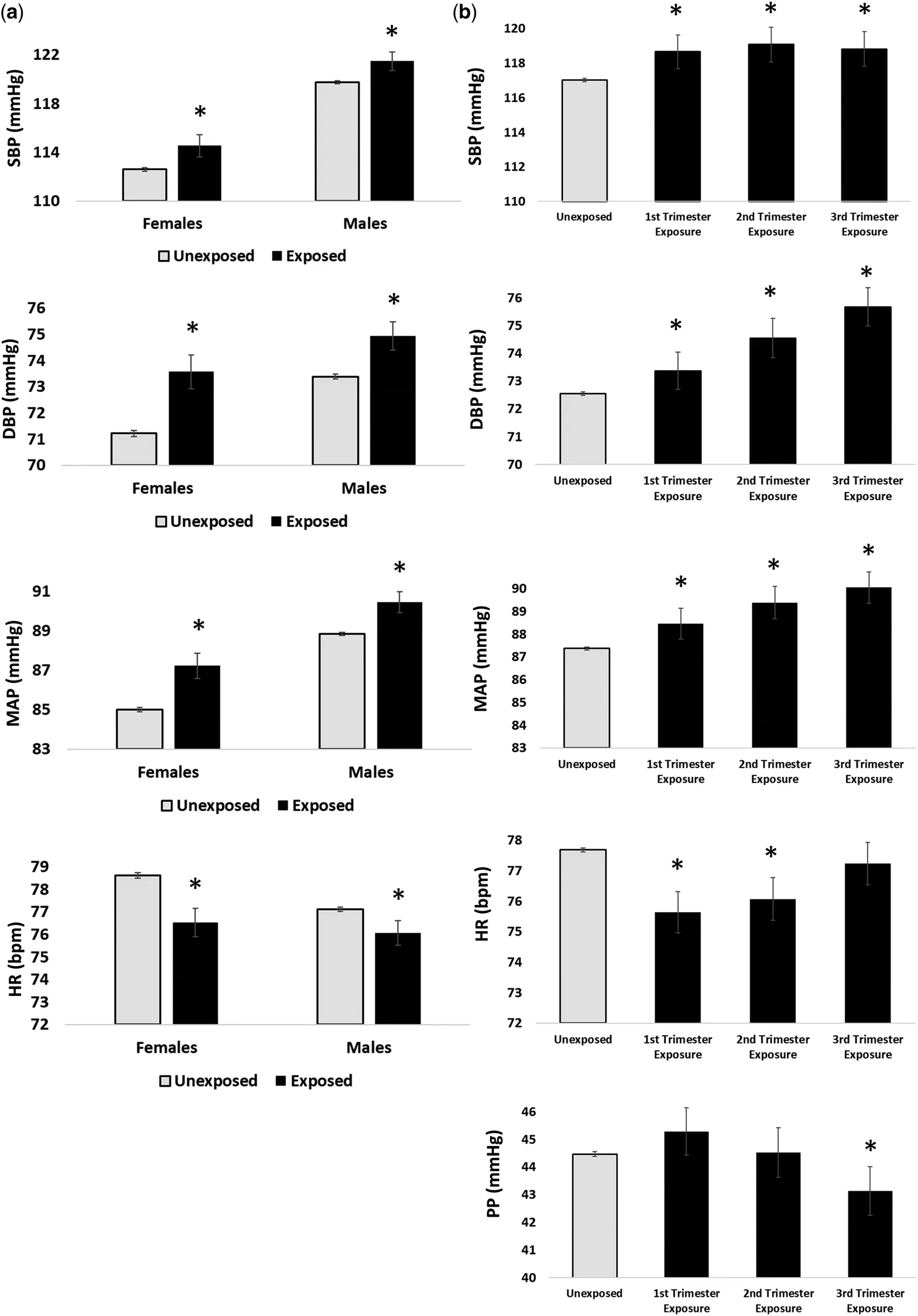
Cardiovascular measurements of offspring whose mothers were exposed or unexposed to the 1967 Six-Day War while pregnant. (a) Estimated marginal means adjusted for all covariates in the regression models in SBP, DBP, MAP, and HR in adolescent female and male offspring. (b) Estimated marginal means adjusted for all covariates in the regression models in SBP, DBP, MAP, HR, and pulse pressure (PP) in young adult offspring given the time of exposure to the war during pregnancy. Error bars denote 95% confidence intervals; asterisks denote *P* < .05 in comparison to the unexposed group; *y*-axes begin at the lowest quartile of observed values.

The results of the adjusted linear regressions, presented in Table 2, indicate that prenatal exposure to acute maternal stress is associated with offspring SBP, DBP, mean arterial pressure (MAP), and HR, overall and in both sexes separately. Based on these models, the estimated marginal mean values adjusted for all regression confounders were calculated. Figure 1a shows that the SBP, DBP, and MAP were higher and the HR was lower among offspring who were prenatally exposed to acute maternal stress. While the effects were consistently observed in both males and females, for DBP and HR, the effects tended to be larger in females (*P* = .023 and *P* = .003 for the interactions, respectively). Conversely, differences in body mass index (BMI) levels, pulse pressure (PP), weight, or height between exposed and unexposed offspring did not reach statistical significance in the adjusted models. Importantly, the introduction of maternal health conditions before or during pregnancy (i.e. pre-eclampsia, diabetes, gestational diabetes, and heart disease) into the models had a negligible effect on the observed associations with BP (Supplementary Table S5). Similarly, when we restricted the non-exposed comparison group to a matched period of ≤3 years following or prior to the war (1964–70) or when we restricted the war-exposure period to 6 days only, the BP regression coefficients remained similar (Supplementary Tables S6 and S7). Finally, when inverse probability weighting (IPW) was used to account for exemption from military service, the results remained nearly unchanged, reducing the likelihood that the findings are driven by selection bias (Supplementary Table S8).

**Table 2:** Overall and sex-specific associations of prenatal exposure to war-induced acute maternal stress with offspring cardiovascular and anthropometric outcomes at age 17 years.

Outcome variable	Total group	Coefficient	95% CI	*P* value	*Interaction with sex *P* value
	(*n* = 60 351)				
**SBP**		1.853	1.149, 2.560	<.001	.639
**DBP**		1.893	1.401, 2.385	<.001	.023
**PP**		–0.040	–0.665, 0.585	.900	.209
**MAP**		1.880	1.389, 2.370	<.001	.081
**HR**		–1.394	–1.883, –0.905	<.001	.003
**Weight**		–0.277	–0.859, 0.305	.351	.210
**Height**		0.025	–0.352, 0.401	.898	.221
**BMI**		–0.103	–0.285, 0.078	.265	.562
**Variable**	**Females**	**Coefficient**	**95% CI**	***P* value**	
	(n = 22 970)				
**SBP**		1.952	1.010, 2.893	<.001	
**DBP**		2.351	1.696, 3.006	<.001	
**PP**		–0.399	–1.233, 0.434	.347	
**MAP**		2.218	1.564, 2.872	<.001	
**HR**		–2.085	–2.737, –1.433	<.001	
**Weight**		0.053	–0.722, 0.828	.893	
**Height**		0.224	–0.275, 0.723	.380	
**BMI**		–0.055	–0.297, 0.187	.657	
**Variable**	**Males**	**Coefficient**	**95% CI**	***P* value**	
	(n = 37 381)				
**SBP**		1.714	0.914, 2.514	<.001	
**DBP**		1.549	0.993, 2.106	<.001	
**PP**		0.165	–0.543, 0.873	.648	
**MAP**		1.604	1.049, 2.160	<.001	
**HR**		–1.049	–1.604, –0.495	<.001	
**Weight**		–0.470	–1.128, 0.189	.162	
**Height**		–0.106	–0.530, 0.318	.625	
**BMI**		–0.131	–0.337, 0.075	.213	

Each row represents a separate linear regression model fitted to examine the association between prenatal exposure to acute stress (exposed versus unexposed as the reference) and a specific cardiovascular outcome. All models were adjusted for parental age, parental education, parental country of origin, socioeconomic status, years since mother immigrated to Israel, number of siblings, offspring birthweight, season, and offspring birth year. Offspring biological sex was adjusted for in the overall models only. CI: 95% confidence interval; PP: pulse pressure; MAP: mean arterial pressure; BMI: body mass index. Overall estimates are based on models without interaction with sex. Sex-specific estimates and the interaction *P* value were derived from models that included the interaction term.

To investigate the potential timing effect, we examined trimester-specific associations with cardiovascular outcomes in offspring (Table 3 and Figure 1b). The levels of SBP, DBP, and MAP were higher among offspring exposed *in utero* at any one of the trimesters compared with those who were unexposed. Testing for the trend further showed that the associations with DBP and MAP were in fact getting stronger throughout the trimesters (*P*_trend_ < .001, and *P*_trend_ = .001, respectively). In contrast, the association with HR was diminishing during pregnancy, being the strongest in the first trimester [*B *= –2.01, 95% confidence interval (CI): –2.67, –1.33] and becoming non-significant in the third (*P*_trend_ < .001). Lastly, as opposed to the null findings from the overall analysis with PP, trimester-specific associations were observed regarding prenatal exposure that occurred during the third trimester of pregnancy (*B *= –1.31, 95% CI: –2.21, –0.42, *P*_trend_ < .001).

**Table 3:** Trimester-specific associations of prenatal exposure to war-induced acute maternal stress versus unexposed pregnancies with offspring cardiovascular and anthropometric outcomes at age 17 years.

Variable	Trimester	Coefficient	95% CI	*P* value	*P*_heterogeneity_	*P*_trend_
**SBP**	1	1.640	0.659, 2.621	.001	.703	.887
	2	2.169	1.141, 3.198	<.001		
	3	1.847	0.833, 2.861	<.001		
**DBP**	1	0.861	0.178, 1.54	.013	≤.001	<.001
	2	2.038	1.322, 2.654	<.001		
	3	3.161	2.455, 3.866	<.001		
**PP**	1	0.779	–0.089, 1.648	.079	<.001	<.001
	2	0.131	–0.778, 1.041	.777		
	3	–1.313	–2.211, –0.417	.004		
**MAP**	1	1.121	0.439, 1.802	.001	.002	.001
	2	2.082	1.368, 2.796	<.001		
	3	2.723	2.019, 3.427	<.001		
**HR**	1	–2.007	–2.686, –1.327	<.001	.001	<.001
	2	–1.551	–2.263, –0.838	<.001		
	3	–0.416	–1.118, 0.286	.245		
**Weight**	1	–0.049	–0.860, 0.762	.906	.731	.608
	2	–0.460	–1.310, 0.389	.248		
	3	–0.404	–1.242, 0.434	.345		
**Height**	1	–0.068	–0.592, 0.455	.799	.828	.740
	2	0.145	–0.403, 0.694	.604		
	3	0.039	–0.502, 0.579	.888		
**BMI**	1	–0.004	–0.257, 0.249	.976	.475	.531
	2	–0.211	–0.476, 0.054	.119		
	3	–0.139	–0.400, 0.122	.298		

Each three-row block represents a separate linear regression model fitted to examine the association between the timing of prenatal exposure to acute stress (i.e. exposure during first, second, or third trimester versus unexposed as the reference) and a specific cardiovascular outcome. All models were adjusted for parental age, parental education, parental country of origin, socioeconomic status, years since mother immigrated to Israel, number of siblings, offspring birthweight, season, offspring birth year, and biological sex. *P*_heterogeneity_ was evaluated through an F-test including trimesters as categories and *P*_trend_ was assessed through the inclusion of trimesters as ordinal values.

## Discussion

Our findings demonstrate that prenatal exposure to a well-defined war-induced acute maternal psychosocial stress was associated with differences in BP measured at age 17 years. SBP, DBP, and MAP were higher and HR was lower in adolescent offspring exposed prenatally to maternal acute stress compared with non-exposed offspring. Offspring body-size traits, however, were unrelated to this stress exposure. All associations were present after controlling for multiple potential confounders. The findings also indicated that differences in the levels of BP between exposed and unexposed offspring tended to be slightly higher in females than in males. Importantly, long-term follow-up studies have linked similar differences in BP in young adults as predictors of premature cardiovascular disease 28 years later [29], suggesting that the observed differences in BP are likely to have meaningful clinical significance. Previous studies using the same cohort have shown that the same prenatal stress exposure was associated with a substantially increased incidence of hospital admission for schizophrenia [26] and mood disorders [27], further emphasizing the impact of exposure to this type of acute stress in pregnancy.

In comparison, in the Generation R study (Netherlands) [10], parental general psychological distress assessed by questionnaires did not affect childhood BP levels and carotid–femoral pulse wave velocity after adjustment for potential confounders. Another study from the Raine cohort that examined stressful life events [11] showed rather opposing findings in which the addition of each prenatal stressor was associated with increased offspring BMI (beta = 0.37 kg/m^2^) but with reduced offspring SBP by 0.66 mmHg. Maternal stress also predicted a lower likelihood of systolic pre-hypertension (SBP ≥ 120 mmHg) in the Avon Longitudinal Study of Parents and Children and was negatively associated with DBP, although not with SBP, in offspring aged 10–12 years [12].

As for the null associations observed in our study with body-size variables, whether or not trauma affected body-weight measures in other studies is also inconsistent. In a Danish cohort, 119 908 youngsters, including 4813 who were born to bereaved mothers (due to the death of a close relative), had similar median BMI levels but a higher incidence of overweight [17]. However, in a systematic review of human studies, acute exposure to prenatal famine was positively related to obesity-related traits during adulthood in women but not in men [30]. Another systematic review of observational studies found that only 8 out of 15 studies have shown a positive association between maternal exposure to stress during pregnancy and obesity-related traits in offspring during childhood [31].

The discrepancies between our findings and those of prior studies (e.g. Supplementary Table S1) likely reflect key methodological and conceptual differences. Unlike studies relying on self-reported or chronic stress, our exposure captures a discrete, time-limited acute stressor, reducing misclassification and confounding by individual perception or behavior. Differences in the timing of the exposure during the pregnancy, as our results suggest trimester-specific effects, may also have contributed. Additionally, variation in the outcome assessment, including age at follow-up and measurement protocols, may limit comparability. Residual confounding from stress-related maternal behaviors (e.g. smoking, diet) may have further influenced the associations. There are several biological mechanisms that could theoretically explain the association observed in our study between stress *in utero* and offspring elevated BP. For example, perturbation of the hypothalamic–pituitary–adrenal (HPA) axis [32] could have impacted the “set points” for the physiologic feedback loops (e.g. leptin, insulin, cortisol) [33], influencing sensitivity to stress [34]. In pregnancy, stress can influence the maternal HPA axis, leading to increased levels of cortisol that may transfer to the fetus. Although some amount of the cortisol secreted by pregnant women is inactivated by 11beta-hydroxysteroid dehydrogenase-2 (11β-HSD-2) in the placenta, particularly in early gestation, some maternal cortisol crosses into the fetus [35]. Additionally, animal studies have shown that prenatal exposure to glucocorticoids is associated with BP at birth, with adult microvascular dysfunction, and with alterations in the renin–angiotensin system [36, 37]. Epigenetic changes within the fetal HPA axis that compromise adrenal angiotensin II type 1b receptor and hypothalamic glucocorticoid receptor function could serve as a potential underlying molecular mechanism [38]. Such an indirect influence of maternal stress on the set point of the fetal HPA and adrenomedullary stress-regulatory systems may contribute to altered offspring physiological responses to stress in later life [39, 40].

Notably, our work also demonstrates that the period of gestation in which mothers were exposed to war-induced acute stress does matter. DBP and MAP tended to be higher and HR was lower with increasing trimesters of pregnancy during the war. In previous analyses of the JPS cohort, the incidence of schizophrenia was raised in offspring who were exposed to the acute stress in the second month of fetal life and mood disorders were substantially increased in offspring exposed during the first trimester of pregnancy [26, 27].

Several studies have argued for the importance of critical windows during development. In the Dutch famine study, offspring born to mothers who were exposed to the famine during late gestation had low birthweight and lower rates of obesity during adult life [41]. Yet, those exposed during early gestation experienced elevated rates of obesity, altered lipid profiles, and cardiovascular disease while those exposed in mid-pregnancy exhibited reduced renal function [16]. Another study reported an association between psychosocial stress measured via questionnaires during the first week of the second trimester and BP in offspring aged 5–7 years [42]. However, the stress measured during this single time point is not necessarily limited to the second trimester. Typically, the first trimester is considered the trimester with the highest fetal vulnerability to any transient exposure such as trauma or diet due to the development of critical basal systems, such as the formation of the central nervous system [43, 44], potentially explaining the stronger association obtained in HR during early pregnancy. However, the total nephron number—a key parameter known to be associated with hypertension—is most rapidly developed in the third trimester [45], in line with the associations observed with BP during late pregnancy.

Our study also demonstrated some sex differences in the association between maternal stress and BP variables. We previously found that the raised incidence of schizophrenia for those who were exposed to war-induced acute stress during the second month of fetal life was much greater in females than in males [26]. Similarly, in the current study, we also found the association of maternal stress with adjusted BP variables and HR to be more prominent in females compared with males. This corresponds to other studies showing greater effects of prenatal famine on obesity, cancer, and cardiovascular mortality in women [20, 21, 46]. One possible explanation is that female fetuses may be more sensitive to *in utero* stress exposure through sex-specific placental adaptations and differential programming of the HPA axis, which can lead to altered cardiovascular regulation [47]. However, in other studies with different outcome measures, larger effects are sometimes found among men compared with women [48–51].

The strengths of our study stem from the large perinatal population-based birth cohort, with valid assessments of cardiovascular measurements at follow-up 17 years after birth and an ability to control for potential confounders. Furthermore, our study is particularly distinctive given that it relies on a single short-lived but very impactful acute episode of war-induced stress, which entails a psychosocial stressor that is not accompanied by any environmental disruption, such as famine, displacement, or toxic exposures. The stressor thus did not have a chronic component that would be hard to disentangle, as is often the case in other scenarios. This unique transient setting also enabled us to analyse the effect of specific gestation periods. One limitation of our study relates to the absence of information on the exact length of gestation. If maternal stress were to lead to an excess of preterm births in vulnerable pregnancies, then there might be a bias in the estimation of gestational age and gestations might have been more advanced than we have estimated. However, in our data, there was no significant difference in birth weight between the exposed and unexposed groups (means of 3302 and 3289 grams, respectively; see also Table 1), suggesting that differences in preterm birth are less likely in our data. Another limitation is that data from age 17 years were not available for all offspring from the original cohort. While this may warrant cautious interpretation of the results among females, it is rather unlikely that exemption prior to the examination is related to either the exposure or the outcomes studied and therefore is not expected to have substantially affected the estimated associations. This assumption is further supported by the analyses utilizing IPW, which produced similar results. Finally, while similar differences in BP in young adults (aged 25 years) were robust predictors of premature cardiovascular disease [29], follow-up studies are needed to confirm the extent to which the differences observed in BP levels translate into clinical endpoints (e.g. heart disease or stroke) in our cohort.

## Conclusions

This study supports the hypothesis that a transient stressful period of armed conflict occurring during fetal life is associated with higher BP levels in adolescence, whereas exposure to this stressor was unrelated to the development of adolescent obesity. Consistent with the developmental determinants of health, this may act through different mechanisms or over different periods. The intergenerational effect of war and trauma is a significant question, more so in light of the catastrophic exposures accompanying war and civic unrest experienced worldwide and that are expected to be exacerbated due to climate change. Understanding the relationship between fetal exposure to stress during sensitive points in gestation and subsequent cardiovascular disease risks may aid in developing interventions to reduce the negative effects of early traumatic events on the health of future generations.

## Ethics approval

The study conforms to the ethical guidelines of the 1975 Declaration of Helsinki and was approved by the Institutional Review Board of the Hadassah-Hebrew University Medical Center.

## Supplementary Material

dyag111_Supplementary_Data

## Data Availability

All data necessary to assess the findings of this study are provided in the tables within the manuscript and Supplementary data. Additional individual-level data are subject to ethical restrictions and cannot be publicly shared, in accordance with the data-access policies of the JPS cohort.
