# Supplementary material for: Acute maternal stress in pregnancy and cardiovascular risk factors in adolescent offspring: a birth-cohort study
Source: Int J Epidemiol. 2026 Jul 23;55(4):dyag111. doi: 10.1093/ije/dyag111 (PMC13394487; doi:10.1093/ije/dyag111)
Supplement: dyag111_Supplementary_Data [file dyag111_supplementary_data.zip › ije-2025-01-0075-File004.docx]

**Supplementary Material**

Supplementary Methods: pages 1-3

Supplementary Figure S1: page 4

Supplementary Tables S1-S8: pages 5-13

**Supplementary Methods**

**Study Variables**

Exposure to prenatal war-induced acute maternal stress was defined using May 19 to June 11 as the war period, or only to the six days of the war in a sensitivity analysis (June 5 to June 10). Gestational month at the time of the war was determined by first estimating the date of conception (by subtracting the average pregnancy length of 266 days from the birth date), and then counting the number of days from conception until the onset of the war. Prenatal exposure was incorporated into the models in two ways: (1) dichotomously, as a binary variable indicating whether the mother was pregnant during the Six-Day War (yes/no, representing exposed and unexposed, respectively); and (2) categorically, based on the timing of exposure, with the war occurring during the 1st, 2nd, or 3rd trimester versus unexposed (each participant included in one group only). The unexposed group included all births occurring before the war or that were conceived after its conclusion.

Cardiovascular and anthropometric outcomes in offspring at age 17 included height (without shoes to the nearest centimeter), body weight (with light clothes to the nearest kilogram), systolic and diastolic blood pressure (SBP and DBP, respectively), and heart rate (HR) measured once in the right arm, in sitting position, with a mercury sphygmomanometer.

Based on these measurements the following additional outcomes were calculated: Body mass index (BMI) calculated by dividing weight (kg) by height squared (m^2^); pulse pressure (PP) estimated as the difference between SBP and DBP; and mean arterial pressure (MAP) defined as the sum of two-thirds of DBP and one-third of SBP. All outcome variables were treated as continuous. It is noteworthy that the outcome measures used in this study were not obtained in a research setting, but rather through a process of clinical assessments related to the induction to the army. This could have some implications in terms of validity. However, previous studies from the Jerusalem Perinatal Study (JPS) demonstrated robust associations between various early life exposures and measurements obtained from the army (e.g. (1-5)). These results are in line with findings from studies in other populations reinforcing the validity of the outcome measurements.

Demographic and socioeconomic characteristics of parents obtained at offspring birth, included data on offspring biological sex (male/female) and birthweight (grams; continuous). The following parental characteristics were included in the analyses as potential confounders: maternal and paternal age at offspring’s birth (years; continuous); maternal and paternal country of birth, or if born in Israel, that of the maternal grandfather (categorized as: Israel, North Africa, Middle East, and Europe/America); maternal and paternal education level (years; continuous); a scale representing socio-economic status (SES, categorized from 1=highest to 3=lowest) based on father’s occupation; maternal parity (number of previous children categorized as 0, 1, or >2); years elapsed since mother immigrated to Israel and until offspring birth (years; continuous, or age at offspring birth for Israel-born mothers). Other characteristics related to the offspring included birth year (categorical, ranging between 1964 and 1976 or up to 1970 in a sensitivity analysis with a restricted comparison group of up to three-years before or after the war), and birth season in four categories. Furthermore, in additional sensitivity analyses we included maternal health conditions and pregnancy complications identified before or during her pregnancy in the birth cohort data (i.e., pre-eclampsia, diabetes, gestational diabetes and heart disease).

**Statistical Analyses**

Multiple linear regressions were used to estimate associations of prenatal stress with offspring outcomes, adjusted for potential confounders. Models were fitted in all offspring as well as separately for males and females. Estimates are reported as regression coefficients (*B*) with 95% confidence intervals (CIs). To explore the effect of timing of prenatal stress, the categorical exposure variable was introduced into the models, and F-tests were conducted to assess heterogeneity of the coefficients between trimester-specific as categories and as ordinal values to assess a general trend. Estimated marginal means were also calculated to represent the outcome means adjusted for all confounders. Interactions between the war exposure and offspring biological sex on outcomes in offspring were examined using multiplicative terms.

Additionally, we used inverse probability weighting (IPW) to mitigate the effect of selection bias due to exemption from military service prior to medical examinations^[[1]](#footnote-1)^. Inverse probabilities were obtained from a logistic regression with non-missingness in ‘offspring BMI at age 17’ variable as the dependent variable and four independent variables: offspring sex, low SEP, low birthweight and religiosity (defined by whether the father was a Yeshiva student or a rabbi), analyzed as dichotomous variables. Weight diagnostics, including effective N=56,800 (compared with unweighted N=61,237), indicated that the weighting performed well. These inverse probabilities were applied as probability weights in the analysis and variance estimates were computed using the robust (sandwich) variance estimator.

Statistical analyses were carried out with STATA version 18.0 (Stata Corp, College Station, TX) and SPSS statistics version 18.0.0. Two-tailed P values < 0.05 were considered significant.

Supplementary Methods References

1. Avgil Tsadok M, Friedlander Y, Paltiel O, Manor O, Meiner V, Hochner H, et al. Obesity and blood pressure in 17‐year‐old offspring of mothers with gestational diabetes: Insights from the Jerusalem Perinatal Study. Journal of Diabetes Research. 2011;2011(1):906154.

2. Dior UP, Karavani G, Bursztyn M, Paltiel O, Calderon-Margalit R, Friedlander Y, et al. Birth Weight and Maternal Body Size as Determinants of Blood Pressure at Age 17: Results from the Jerusalem Perinatal Study Cohort. Matern Child Health J. 2021;25(1):162-71.

3. Dior UP, Lawrence GM, Sitlani C, Enquobahrie D, Manor O, Siscovick DS, et al. Parental smoking during pregnancy and offspring cardio-metabolic risk factors at ages 17 and 32. Atherosclerosis. 2014;235(2):430-7.

4. Hochner H, Friedlander Y, Calderon-Margalit R, Meiner V, Sagy Y, Avgil-Tsadok M, et al. Associations of maternal prepregnancy body mass index and gestational weight gain with adult offspring cardiometabolic risk factors: the Jerusalem Perinatal Family Follow-up Study. Circulation. 2012;125(11):1381-9.

5. Shapiro I, Youssim I, Paltiel O, Calderon-Margalit R, Manor O, Friedlander Y, et al. Perinatal exposures and adolescence overweight: The role of shared maternal-offspring pathways. Atherosclerosis. 2024;389:117438.

**Supplementary Figure S1**

Supplementary Figure S1: The number of individuals in the Jerusalem Perinatal Study birth cohort with or without military draft examination data, and the number of those who were prenatally exposed or unexposed to war-induced acute maternal stress

**Supplementary Tables**

Supplementary Table S1: Comparison of key publications testing offspring outcomes given exposure to perinatal stress and their primary findings

| **Study (Reference)** | **Birth Cohort (Period)** | **Total N (Exposed)** | **Stress Exposure** | **Exposure Time** | **Offspring Age** | **Main BP Outcomes** | **Main Body Weight Outcomes** | **Covariates** |
| --- | --- | --- | --- | --- | --- | --- | --- | --- |
| Generation R study (10) | Netherlands | 4,831 | Psychological distress questionnaires | 2nd trimester | 6 years | Non significant after covariate adjustments | Not measured as an outcome | Maternal age, parity, educational level, smoking, ethnicity, pre-pregnancy body mass index (BMI) and paternal ethnicity; Date of birth, child sex, gestational age at birth and birth weight; Breastfeeding; offspring BMI |
| Raine Study(11) | Western Australia (1989-1992) | 957 | Stressful life events | 2nd trimester | 20 years | Reduced offspring SBP | Increased offspring BMI | ppBMI before pregnancy; maternal height; Birth weight; Maternal education; education; offspring current stress; Maternal hypertension; preeclampsia; maternal smoking; offspring smoking, alcohol consumption, and female hormonal contraceptive usage; Interaction with offspring BMI |
| Avon Longitudinal Study (12) | Avon, UK (1991-1992) | 4,318 | Anxiety questionnaires | 2nd, 3rd trimesters | 10-11 years | Lower likelihood of systolic pre-hypertension; lower DBP | Not measured as an outcome | Maternal age; gestational age; birthweight; offspring sex; ppBMI; Parity; Paternal occupational social class; Maternal ethnicity; smoking; alcohol; Age of child at assessment; Child BMI in some models |
| Danish National Cohort Study (17) | Denmark (1976 -1993) | 119,908 (4,813) | Bereaved mothers due to death of a close relative | Pre-conception to birth | ~18 years | Not measured | Similar BMI, yet Increased rates of overweight | Parity; gestational age; maternal age; cohabitating status at birth; maternal socio-economic factors; education; income; birth weight included as an intermediate factor |
| Doyle et al., 2000 (8) | Melbourne (1977-1982) | 177 (89) | Antenatal corticosteroid therapy | Late 2nd/Early 3rd | 14 years | Higher SBP & DBP | Not significant | Gestational age; birthweight; birthweight S.D; maternal hypertension in pregnancy; gender; need for assisted ventilation; pneumothorax or other airleak; durations of assisted ventilation and oxygen therapy; weight S.D and height S.D. scores at age 14 |
| Fan et al, 2016 (9) | China (Not stated) | 216 | Anxiety questionnaires | 1st; 2nd; 3rd trimesters | 7-9 years | Higher SBP & DBP & HR | Not measured | Birth weight |
| Amsterdam Born Children and their Development study (19) | Amsterdam (2003-2004) | 2,968 | Anxiety questionnaires | 2nd trimester | 5-7 Years | Higher SBP & DBP & MAP (for exposed to 3-4 psychological stressors | Not measured as an outcome | Maternal age; ethnicity; ppBMI; educational level; smoking; alcohol consumption; pre-existing conditions (hypertension, diabetes, hypothyroidism, hyperthyroidism and epilepsy); Parity; gestational age at birth; birth weight; sex; Maternal (family) hypertension; Paternal (family) hypertension; child BMI |
| Danish and Swedish registries (18) | Denmark & Sweeden (1973-2016) | 6,758,560 (8,664) | Bereaved mothers due to death of a close relative | Pre-conception to birth | first 5 decades of life | increased risk of ischemic heart disease when loss in 3rd trimester | Not measured | maternal country of origin; marital status; highest educational level; age at delivery; parity; smoking; BMI) in early pregnancy; diseases before delivery (diabetes, hypertensive disease, psychiatric disorders); family history of CVD; Offspring characteristics: sex, calendar year of birth; gestational age; birth weight; diagnoses of congenital heart diseases; preterm birth; small for gestational age birth. |

Supplementary Table S2. Comparison of participants from the original cohort with and without army data.

| **Variable** | **Categories/scale** | **Army Data Available (N=61,237)** | **Army Data Unavailable (N=27,151)** | **SMD** |
| --- | --- | --- | --- | --- |
| Sex % | Male | 61.95 | 29.29 | 0.694 |
| Birth weight (kg) | *continuous* | 3.29±0.50 | 3.24±0.51 | 0.094 |
| Paternal age (yrs) | *continuous* | 31.5±6.6 | 31.5±7.3 | 0.006 |
| Maternal age (yrs) | continuous | 27.6±5.5 | 27.7±5.9 | 0.020 |
| Paternal origin % |  |  |  |  |
|  | Israel | 13.6 | 24.0 | 0.269 |
|  | North Africa | 22.6 | 16.8 | 0.147 |
|  | West Asia | 33.3 | 24.0 | 0.207 |
|  | West Europe /Other | 30.5 | 35.2 | 0.102 |
| Maternal origin % |  |  |  |  |
|  | Israel | 12.7 | 22.3 | 0.255 |
|  | North Africa | 24.4 | 17.9 | 0.160 |
|  | West Asia | 31.7 | 23.6 | 0.182 |
|  | West Europe /Other | 31.2 | 36.2 | 0.105 |
| Paternal education (yrs) | *continuous* | 11.2±4.3 | 11.2±4.6 | 0.010 |
| Maternal education (yrs) | *continuous* | 10.5±4.2 | 9.7±4.4 | 0.164 |
| SES % |  |  |  |  |
|  | Low | 25.6 | 25.4 | 0.005 |
|  | Medium | 41.3 | 28.4 | 0.273 |
|  | High | 33.1 | 46.2 | 0.272 |
|  |  |  |  |  |
| Years since Immigration (yrs) | *continuous* | 20.2±8.8 | 19.7±9.7 | 0.054 |

As expected, when comparing the samples with and without army data, the main imbalance is in the proportion of females in the samples (38% versus 70%, respectively). Other, more moderate imbalances observed in socio economic status (SES) and parental country of origin are consistent with the underrepresentation of ultra-orthodox females of Western European origin in the sample with army data. SMD denotes the standardized mean difference.

Supplementary Table S3. Offspring unadjusted cardiovascular and anthropometric outcomes at age 17 by prenatal exposure to war-induced acute maternal stress and offspring biological sex.

| **Total Group** | **Variable** | **Unexposed**  (n=58,766) | **War exposure trimester** | | |
| --- | --- | --- | --- | --- | --- |
|  |  |  | **1^st^ trimester**  (n=675) | **2nd trimester**  (n=878) | **3rd trimester**  (n=918) |
|  | SBP (mmHg) | 117.0±12.6^*^ | 119.4±12.9 | 118.2±12.8 | 117.4±12.7 |
|  | DBP (mmHg) | 72.6±8.4^*^ | 73.6±7.4 | 73.4±8.6 | 74.2±7.9 |
|  | PP (mmHg) | 44.4±10.9^*^ | 45.9±11.8 | 44.9±12.0 | 43.2±11.0 |
|  | MAP (mmHg) | 87.4±8.6^*^ | 88.5±7.8 | 88.3±8.5 | 88.6±8.2 |
|  | HR (bpm) | 77.6± 8.5 | 77.3±7.0 | 77.2±7.3 | 78.1±8.1 |
|  | Weight (kg) | 61.3±10.7^*^ | 60.9±10.4 | 60.4±10.2 | 60.2±9.9 |
|  | Height (cm) | 169.6±8.5^*^ | 169.2±8.3 | 168.9±8.5 | 169.0±7.9 |
|  | BMI (kg/m^2^) | 21.6±3.1 | 21.2±3.0 | 21.1±2.8 | 20.0±2.9 |
|  |  |  |  |  |  |
| **Males** | **Variable** | **Unexposed**  (n=36,411) | **War exposure trimester** | | |
|  |  |  | **1^st^ trimester**  (n=424) | **2nd trimester**  (n=524) | **3rd trimester**  (n=579) |
|  | SBP (mmHg) | 119.8±12.1^*^ | 122.6±12.0 | 120.8±12.8 | 119.8±12.1 |
|  | DBP (mmHg) | 73.4±8.3^*^ | 74.1±7.4 | 73.7±7.9 | 74.9±7.4 |
|  | PP (mmHg) | 46.4±10.9^*^ | 48.5±11.2 | 47.1±12.4 | 44.9±11.3 |
|  | MAP (mmHg) | 88.9±8.2^*^ | 90.2±7.6 | 89.4±7.9 | 89.9±7.6 |
|  | HR (bpm) | 77.1± 8.7 | 77.1±7.0 | 77.2±6.9 | 77.9±8.2 |
|  | Weight (kg) | 64.1±10.7^*^ | 63.5±10.6 | 63.5±10.3 | 62.4±9.8 |
|  | Height (cm) | 173.8±6.9^*^ | 173.2±7.1 | 173.4±7.1 | 172.7±6.6 |
|  | BMI (kg/m^2^) | 21.2±3.1 | 21.1±3.0 | 21.1±2.9 | 20.9±2.9 |
|  |  |  |  |  |  |
| **Females** | **Variable** | **Unexposed**  (n=23,294) | **War exposure trimester** | | |
|  |  |  | **1^st^ trimester**  (n=251) | **2nd trimester** (n=354) | **3rd trimester**  (n=402) |
|  | SBP (mmHg) | 112.5±12.1^*^ | 114.1±12.6 | 114.4±11.7 | 113.2±12.5 |
|  | DBP (mmHg) | 71.2±8.4^*^ | 72.7±7.3 | 72.9±9.5 | 72.8±8.5 |
|  | PP (mmHg) | 41.3±10.0 | 41.4±11.4 | 41.6±10.5 | 40.4±10.0 |
|  | MAP (mmHg) | 84.9±8.6^*^ | 86.5±7.7 | 86.7±9.0 | 86.3±8.8 |
|  | HR (bpm) | 78.5±8.0^*^ | 77.7±7.2 | 77.2±7.7 | 78.4±7.9 |
|  | Weight (kg) | 56.7±8.9 | 56.5±8.2 | 55.8±8.1 | 56.4±9.0 |
|  | Height (cm) | 162.8±6.2 | 162.5±5.3 | 162.1±5.5 | 162.8±5.8 |
|  | BMI (kg/m^2^) | 21.4±3.1 | 21.4±2.9 | 21.2±2.6 | 21.3±3.0 |

Unadjusted means of BP: blood pressure. SBP: systolic BP. DBP: diastolic BP. PP: pulse pressure. MAP: mean arterial pressure. * P-value< 0.01 from one-way analysis of variance joint test of mean values with 3 degrees of freedom. Values are expressed as mean ± SD.

Supplementary Table S4. Offspring unadjusted cardiovascular and anthropometric outcomes at age 17 of those born prior to or conceived after the war.

| **Outcome** | **Born Prior**  **to the War** | **Conceived After the War** |
| --- | --- | --- |
| SBP (mmHg) | 117.4 ± 13.2 | 116.9 ± 12.4 |
| DBP (mmHg) | 72.9 ± 8.6 | 72.5 ± 8.4 |
| PP (mmHg) | 44.6 ±11.1 | 44.4 ± 10.8 |
| MAP (mmHg) | 87.7 ±9.0 | 87.3 ± 8.3 |
| HR (bpm) | 77.4 ± 8.6 | 77.7 ± 8.4 |
| Weight (kg) | 60.3±10.1 | 61.6±10.9 |
| Height (cm) | 169.0±8.1 | 169.8±8.6 |
| BMI (kg/m^2^) | 21.1±2.9 | 21.3±3.2 |

Unadjusted means of BP: blood pressure. SBP: systolic BP. DBP: diastolic BP. PP: pulse pressure. MAP: mean arterial pressure. Values are expressed as mean +- SD.

Supplementary Table S5. Overall associations of prenatal exposure to war-induced acute maternal stress with offspring cardiovascular and anthropometric outcomes at age 17 after including maternal health conditions.

| **Outcome variable** | **Coefficient** | **95% CI** | ***P*-value** |
| --- | --- | --- | --- |
|  |  |  |  |
| SBP | 1.799 | 1.092; 2.506 | <0.001 |
| DBP | 1.846 | 1.354; 2.339 | <0.001 |
| PP | -0.047 | -0.674; 0.579 | 0.882 |
| MAP | 1.831 | 1.339; 2.322 | <0.001 |
| HR | -1.494 | -1.929; -0.949 | <0.001 |
| Weight | -0.277 | -0.859; 0.305 | 0.351 |
| Height | 0.018 | -0.357; 0.393 | 0.926 |
| BMI | -0.103 | -0.285; 0.079 | 0.266 |

Each row represents a separate linear regression model fitted to examine association between prenatal exposure to acute stress (exposed versus unexposed as the reference) and a specific cardiovascular outcome. All models were adjusted for the presence/absence of maternal health conditions (pre-eclampsia, heart disease, diabetes or gestational diabetes), as well as for all the original covariates: parental age, parental education, parental country of origin, socioeconomic status, years since mother immigrated to Israel, number of siblings, offspring birthweight, season, offspring biological sex, and offspring birth year. CI: 95% confidence interval; BP: blood pressure; SBP: systolic BP; DBP: diastolic BP; PP: pulse pressure; HR: heart rate; MAP: mean arterial pressure; BMI: body mass index.

Supplementary Table S6. Overall associations of prenatal exposure to war-induced acute maternal stress with offspring cardiovascular and anthropometric outcomes at age 17 when restricting the non-exposed comparison group to a matched period of up to three years following or prior to the war (1964-1970).

| **Outcome variable** | **Coefficient** | **95% CI** | ***P*-value** |
| --- | --- | --- | --- |
|  |  |  |  |
| SBP | 1.829 | 1.095; 2.563 | <0.001 |
| DBP | 1.774 | 1.284; 2.264 | <0.001 |
| PP | .055 | -0.584; 0.694 | 0.866 |
| MAP | 1.792 | 1.294; 2.291 | <0.001 |
| HR | -1.695 | -2.171; -1.219 | <0.001 |
| Weight | -0.341 | -0.894; 0.213 | 0.228 |
| Height | -0.019 | -0.389; 0.351 | 0.921 |
| BMI | -0.117 | -0.290; 0.056 | 0.186 |

Each row represents a separate linear regression model fitted to examine association between prenatal exposure to acute stress (exposed versus unexposed as the reference) and a specific cardiovascular outcome. All models were adjusted for all the original covariates: parental age, parental education, parental country of origin, socioeconomic status, years since mother immigrated to Israel, number of siblings, offspring birthweight, season, offspring biological sex, and offspring birth year. CI: 95% confidence interval; BP: blood pressure; SBP: systolic BP; DBP: diastolic BP; PP: pulse pressure; HR: heart rate; MAP: mean arterial pressure; BMI: body mass index.

Supplementary Table S7. Overall associations of prenatal exposure to war-induced acute maternal stress with offspring cardiovascular and anthropometric outcomes at age 17 when restricting the exposure war period to only the 6-days (June 5 to Jun10, 1967).

| **Outcome variable** | **Coefficient** | **95% CI** | ***P*-value** |
| --- | --- | --- | --- |
|  |  |  |  |
| SBP | 1.806 | 1.097; 2.515 | <0.001 |
| DBP | 1.875 | 1.381; 2.368 | <0.001 |
| PP | -0.069 | -0.697; 0.559 | 0.830 |
| MAP | 1.852 | 1.359; 2.344 | <0.001 |
| HR | -1.457 | -1.948; -0.965 | <0.001 |
| Weight | -0.271 | -0.854; 0.313 | 0.363 |
| Height | 0.007 | -0.369; 0.383 | 0.972 |
| BMI | -0.096 | -0.279; 0.086 | 0.301 |

Each row represents a separate linear regression model fitted to examine association between prenatal exposure to acute stress (exposed versus unexposed as the reference) and a specific cardiovascular outcome. All models were adjusted for all the original covariates: parental age, parental education, parental country of origin, socioeconomic status, years since mother immigrated to Israel, number of siblings, offspring birthweight, season, offspring biological sex, and offspring birth year. CI: 95% confidence interval; BP: blood pressure; SBP: systolic BP; DBP: diastolic BP; PP: pulse pressure; HR: heart rate; MAP: mean arterial pressure; BMI: body mass index.

Supplementary Table S8. Overall associations of prenatal exposure to war-induced acute maternal stress with offspring cardiovascular and anthropometric outcomes at age 17 using inverse probability weighting (IPW).

| **Outcome variable** | **Coefficient** | **95% CI** | ***P*-value** |
| --- | --- | --- | --- |
|  |  |  |  |
| SBP | 1.792 | 1.029; 2.555 | <0.001 |
| DBP | 1.854 | 1.313; 2.394 | <0.001 |
| PP | -0.061 | -0.772; 0.649 | 0.866 |
| MAP | 1.833 | 1.307; 2.359 | <0.001 |
| HR | -1.686 | -2.168; -1.204 | <0.001 |
|  |  |  |  |
| Weight | -0.309 | -0.880; 0.263 | 0.290 |
| Height | 0.055 | -0.318; 0.428 | 0.773 |
| BMI | -0.127 | -0.307; 0.054 | 0.169 |

Each row represents a separate linear regression model fitted to examine association between prenatal exposure to acute stress (exposed versus unexposed as the reference) and a specific cardiovascular outcome. All models were adjusted for all the original covariates: parental age, parental education, parental country of origin, socioeconomic status, years since mother immigrated to Israel, number of siblings, offspring birthweight, season, offspring biological sex, and offspring birth year. CI: 95% confidence interval; BP: blood pressure; SBP: systolic BP; DBP: diastolic BP; PP: pulse pressure; HR: heart rate; MAP: mean arterial pressure; BMI: body mass index. See supplementary methods for a detailed description of the weighting calculation.

1. It is important to note that the Israeli military differs substantially from settings that rely on voluntary enlistment (or males only programs), where recruits may systematically differ from the general population. This concern is largely not applicable in Israel, given the compulsory nature of service for both males and females. Therefore, the general population is considered the target population of this study. This is true with the caveat that ultra-orthodox females are more likely to be exempt prior to the medical examination. [↑](#footnote-ref-1)
