# Supplementary figures and images for: Acute maternal stress in pregnancy and cardiovascular risk factors in adolescent offspring: a birth-cohort study
Source: Int J Epidemiol. 2026 Jul 23;55(4):dyag111. doi: 10.1093/ije/dyag111 (PMC13394487; doi:10.1093/ije/dyag111)

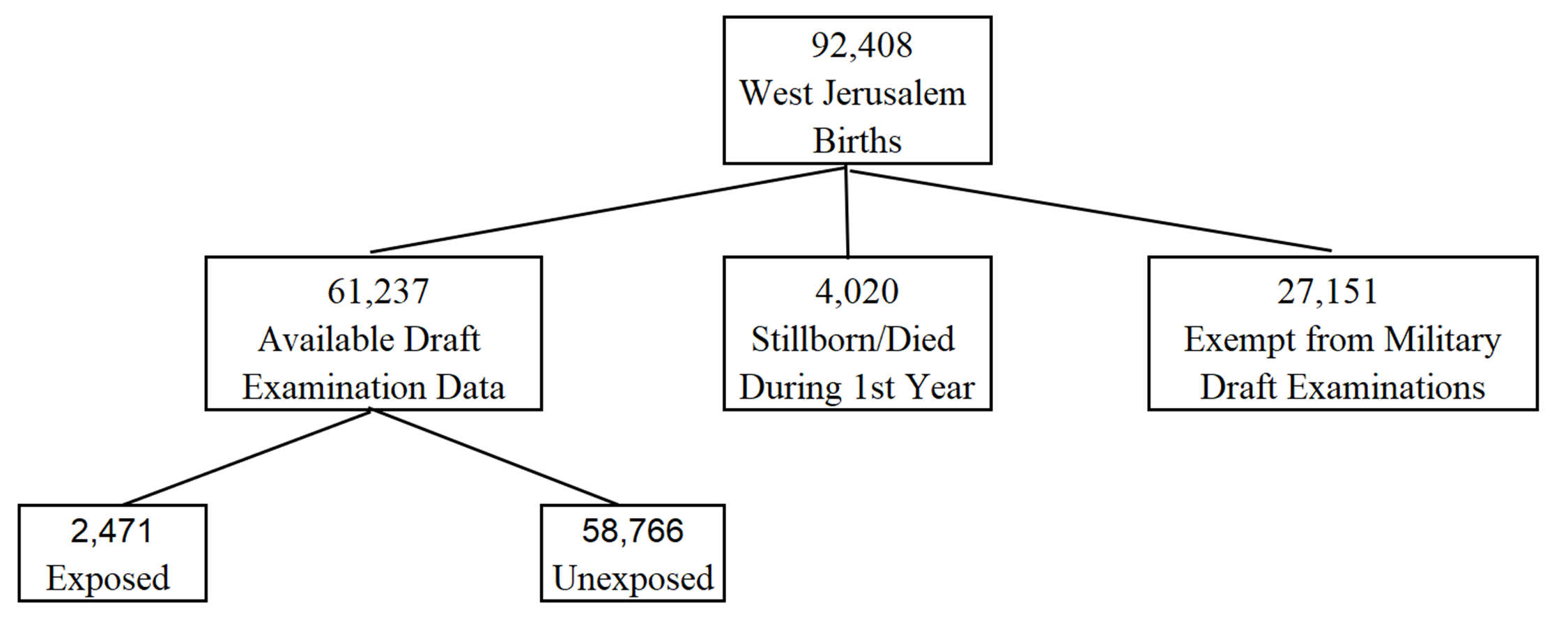

Supplement: dyag111_Supplementary_Data [file dyag111_supplementary_data.zip › ije-2025-01-0075-File005.tif]
